# Progressive Multifocal Leukoencephalopathy Misdiagnosed as a Stroke

**DOI:** 10.1155/crnm/6610404

**Published:** 2026-11-04

**Authors:** Ger Mullan, Stephen Ramsay, Michael Jamison, Martin Harley, Simon Au

**Affiliations:** ^1^ Wellcome-Wolfson Institute for Experimental Medicine Queen’s University Belfast Belfast UK qub.ac.uk; ^2^ Department of Medicine Lagan Valley Hospital Lisburn UK duhs.edu.pk; ^3^ Department of Neurology Ulster Hospital Dundonald UK setrust.hscni.net; ^4^ Department of Neuroradiology Royal Victoria Hospital Belfast UK nhs.uk

**Keywords:** case report, PML, progressive multifocal leukoencephalopathy, stroke

## Abstract

Progressive multifocal leukoencephalopathy (PML) is rare and can be challenging to diagnose. Symptoms can be varied and can mimic stroke phenotypes. Physicians must hold a high index of suspicion in patients with significant PML risk factors (including HIV/AIDS, immunosuppression, lymphoproliferative malignancies and chemotherapy/monoclonal antibody therapies) presenting with progressive neurological symptoms. Here, we describe a 63‐year‐old man who presented with left‐sided weakness, apraxia and neglect on a background of subtle cognitive decline. He had a past medical history of recurrent follicular lymphoma with chemotherapy. He was initially diagnosed as a stroke but deteriorated further and ultimately received a diagnosis of PML. Earlier recognition of this condition will help to facilitate research into this disease.

## 1. Introduction

Progressive multifocal leukoencephalopathy (PML) is a rare demyelinating disease of the central nervous system (CNS) caused by John Cunningham virus (JCV). No evidence‐based treatments are available, and mortality rates are high [1]. Here, we present a case of PML initially misdiagnosed as a stroke. Our case is unique in the diagnostic delay from initial symptom onset and highlights the need for a high index of suspicion in those with relevant risk factors.

## 2. Case Presentation

A 63‐year‐old right‐handed man presented to the emergency department with a reported 4‐day history of progressive left arm weakness and numbness, incoordination and gait disturbance. Through collateral history later, his left‐sided symptoms appeared to have progressed over several weeks. He was admitted under the stroke team. Examination revealed a mild left facial droop, mild left upper limb weakness, four‐limb motor apraxia, reduced left‐sided sensation, left sensory inattention, and mild receptive and expressive dysphasia.

Past medical history included recurrent follicular lymphoma, treated initially with six cycles of O‐CHOP chemotherapy/monoclonal antibody therapy (including obinutuzumab, an anti‐CD20 agent) commenced 2.5 years before symptom onset, with later maintenance obinutuzumab, completed 2 months prior to symptom onset. He had a 40 pack‐year smoking history.

Continuous electrocardiogram monitoring, HbA1c and lipids were normal; HIV was negative. Computerised tomography (CT) of his chest, abdomen and pelvis was unremarkable. CT brain reported an area of low attenuation in the right parietal lobe, possibly in keeping with subacute infarction (see Figure 1). Magnetic resonance imaging (MRI) demonstrated several scattered areas of abnormal signal change primarily involving the subcortical and deep white matter of both cerebral hemispheres, most prominently towards the inferior parietal and posterior temporal lobes on the right side. Subsequent MRI with contrast further demonstrated these findings, without evidence of gadolinium enhancement or diffusion restriction (see Figure 2A). The changes were felt to be suggestive of mature ischaemic changes with postinfarction gliosis, although a wide differential for these appearances was acknowledged. Some of the reported ischaemic change was felt to be in watershed territory. MR angiography showed minor irregularity in the distal branches of the middle cerebral arteries but was otherwise normal. PML and CNS lymphoma were reported as unlikely. Contemporaneous neuroradiology review stated that these appearances were atypical for stroke; differentials of vasculitis, postchemotherapy changes and demyelination were suggested. Vasculitis and connective tissue disease screens were negative. Vascular and haematology consults were reassuring. The admitting team, having considered the radiological findings in light of his clinical presentation made a diagnosis of a right parietal stroke of probable atherothromboembolic origin. He was commenced on antiplatelet therapy and discharged. After initial improvement with rehabilitation, his symptoms continued to progress. A further MRI brain 2 months after initial presentation noted slightly more extensive occipital lobe gliotic change bilaterally but otherwise reported broadly stable gliosis (see Figure 2B), and a basic neurological exam appeared stable.

**FIGURE 1:**
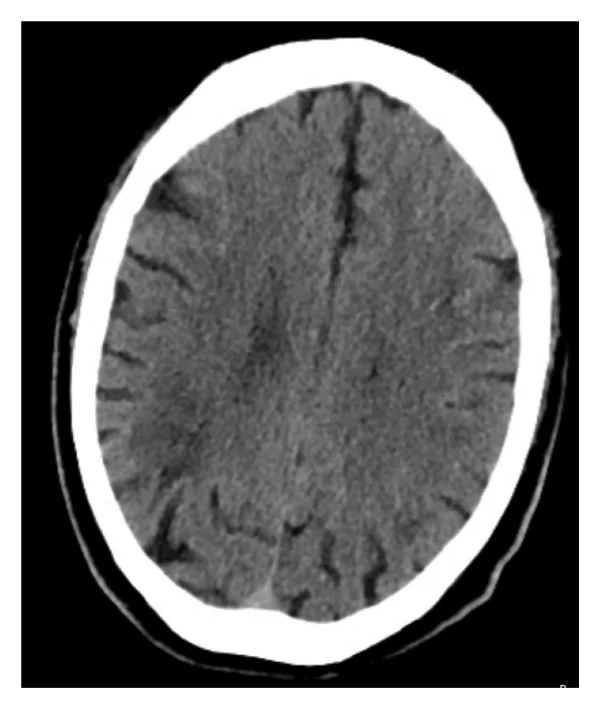
Computed tomography (CT) brain demonstrating area of right parietal hypoattenuation with extension into the gyri.

**FIGURE 2:**
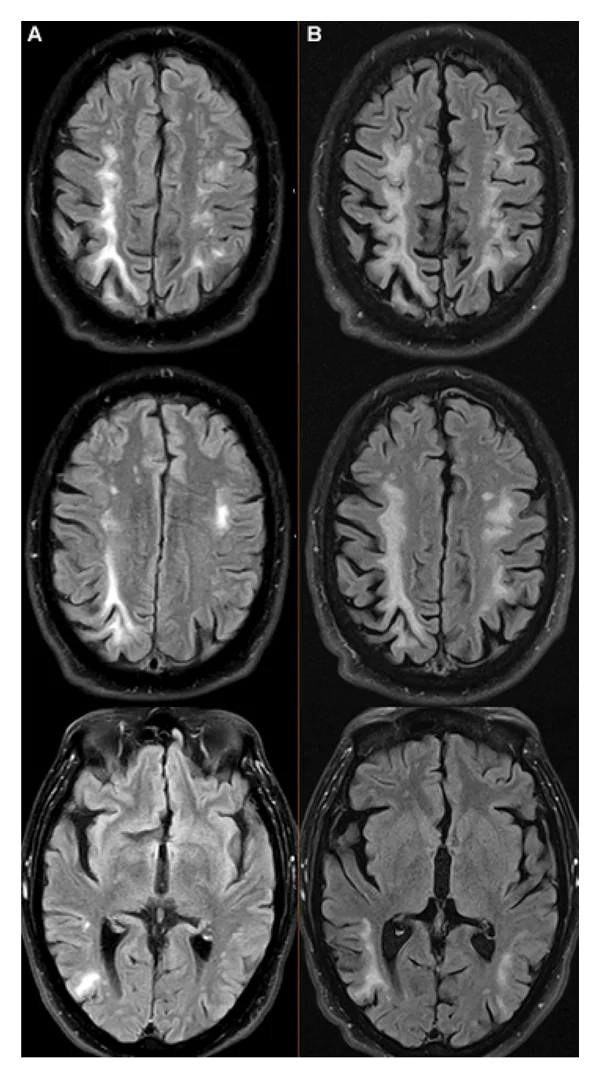
(A) Axial fluid‐attenuated inversion recovery (FLAIR) magnetic resonance imaging (MRI) slices at initial presentation demonstrating demyelination involving the subcortical and deep white matter of both cerebral hemispheres, most prominently towards the inferior parietal and posterior temporal lobes on the right side, and (B) axial T2‐weighted MRI slices 3 months later demonstrating subsequent progression with the absence of both volume loss and encephalomalacia that would be expected in postinfarct gliosis. Subcortical U‐fibre involvement, one of the radiological hallmarks of progressive multifocal leukoencephalopathy (PML), is observed in both studies.

He presented elsewhere a month later with deteriorating mobility. His family described progressive dyspraxia and mentioned paranoid themes of thought content. On reflection, they reported a 6‐month history predating his initial presentation of subtle progressive apraxia and personality change (particularly loss of humour). Examination revealed left facial weakness, left hemiparesis, right upper limb and gait ataxia, anomia, acalculia, emotional lability, perseveration and later disinhibition. A diagnosis of PML was suspected. Routine cerebrospinal fluid (CSF) analysis was unremarkable except for total protein 0.49 g/L. CSF analysis for JCV was positive. He was diagnosed with PML and deteriorated despite mirtazapine treatment. He died two weeks after diagnosis, 12 months after symptom onset.

## 3. Discussion

This case highlights the difficulty in diagnosing PML. Symptoms can be varied based on disease location and can mimic stroke phenotypes [1, 2]. Physicians must hold a high index of suspicion in patients with significant PML risk factors (including HIV/AIDS, immunosuppression, lymphoproliferative malignancies and chemotherapy or specific monoclonal antibody therapies) presenting with progressive neurological symptoms, particularly with multifocal imaging abnormalities [1, 3, 4]. Indeed, Hlaing and Maung describe a separate case of subacute progressive neurological deficit with an initial suspected diagnosis of stroke in which PML was not initially considered as a differential. A positive HIV test in this case widened differentials and subsequently led to a PML diagnosis [2].

Collateral history was key in this case. Initial history from the patient was vague, which likely reflected a degree of higher cognitive impairment. The focus on this patient‐reported history led the treating team to believe that the syndrome was more acute than what it truly was, with CT imaging unable to definitively age the initial lesion. Findings on MRI had a wide differential on neuroradiology review, and a reported short history may have made PML seem an unlikely diagnosis. A later detailed collateral history uncovered subtleties revealing a much longer and more subacute onset than initially thought. This detail, paired with retrospectively acknowledged subcortical U‐fibre involvement in both MRI studies and relevant history of lymphoma and subsequent treatment, would have been strongly suggestive of PML as a differential diagnosis.

Whilst rare, PML incidence may rise in coming years due to increasing use of monoclonal antibody therapy [5]. Of note, there is a documented PML risk with obinutuzumab used in this case. This is highlighted in both the Food and Drug Administration label [6] and the European Medicines Agency Summary of product characteristics [7].

To date, there is no effective treatment for PML [1, 3]. Ultimately, earlier recognition of this condition will help to facilitate research into this disease.

## Funding

This research received funding support from Queen’s University Belfast, for the purposes of article processing charges only.

## Consent

Informed consent for case report publication was obtained from the patient’s next of kin.

## Conflicts of Interest

The authors declare no conflicts of interest.

## Data Availability

The data that support the findings of this study are available from the corresponding author upon reasonable request.
